# Poly[[[diaqua­sodium]-μ_3_-5-carb­oxy-2-ethyl-1*H*-imidazole-4-carboxyl­ato-κ^4^ *N* ^3^,*O* ^4^:*O* ^5^:*O* ^5^] monohydrate]

**DOI:** 10.1107/S1600536811002741

**Published:** 2011-02-02

**Authors:** Shi-Jie Li, Xiao-Tian Ma, Wen-Dong Song, Xiao-Fei Li, Juan-Hua Liu

**Affiliations:** a College of Food Science and Technology, Guangdong Ocean University, Zhanjiang 524088, People’s Republic of China; b College of Science, Guangdong Ocean University, Zhanjiang 524088, People’s Republic of China; c College of Agriculture, Guangdong Ocean University, Zhanjiang 524088, People’s Republic of China

## Abstract

In the title complex, {[Na(C_7_H_7_N_2_O_4_)(H_2_O)_2_]·H_2_O}_*n*_, the Na^I^ atom exhibits a distorted octa­hedral geometry and is six-coordinated in an NO_5_ environment. The equatorial plane is defined by three O atoms and one N atom from two distinct 5-carb­oxy-2-ethyl-1*H*-imidazole-4-carboxyl­ate (H_2_EIDC)  ligands and one coordinated water mol­ecule, and the apical sites are occupied by one carboxyl O atom from one H_2_EIDC ligand and one O atom from the other coordinated water mol­ecule. The Na^I^ atoms are linked by H_2_EIDC ligands, generating an infinite double chain along the *a* axis. These chains are further connected *via* O—H⋯O and N—H⋯O hydrogen bonds into a three-dimensional supra­molecular network.

## Related literature

For the rational design of metal coordination complexes, see: Sava *et al.* (2009 ▶); Lu *et al.* (2010 ▶); Xue *et al.* (2009 ▶). For H_3_IDC complexes with supra­molecular architectures, see: Zou *et al.* (2006 ▶); Li *et al.* (2006 ▶); Sun *et al.* (2005 ▶). For related coord­in­ation polymers based on H_3_EIDC, see: Wang *et al.* (2008 ▶); Zhang *et al.* (2010 ▶).
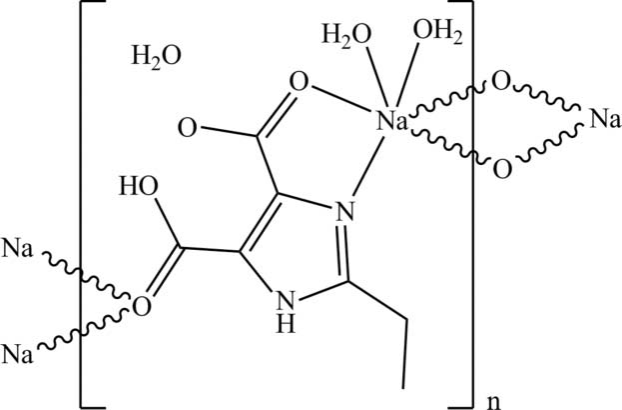


## Experimental

### 

#### Crystal data

[Na(C_7_H_7_N_2_O_4_)(H_2_O)_2_]·H_2_O
*M* *_r_* = 260.18
Monoclinic, 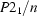

*a* = 8.5231 (8) Å
*b* = 7.0598 (7) Å
*c* = 19.0329 (17) Å
β = 98.880 (1)°
*V* = 1131.51 (18) Å^3^
*Z* = 4
Mo *K*α radiation
μ = 0.17 mm^−1^
*T* = 298 K
0.49 × 0.48 × 0.34 mm

#### Data collection

Bruker SMART 1000 CCD area-detector diffractometer
Absorption correction: multi-scan (*SADABS*; Bruker, 2007 ▶) *T*_min_ = 0.923, *T*_max_ = 0.946
5410 measured reflections
1991 independent reflections
1549 reflections with *I* > 2σ(*I*)
*R*_int_ = 0.043

#### Refinement

*R*[*F* ^2^ > 2σ(*F* ^2^)] = 0.039
*wR*(*F* ^2^) = 0.109
*S* = 1.04
1991 reflections
162 parameters
9 restraints
H atoms treated by a mixture of independent and constrained refinement
Δρ_max_ = 0.33 e Å^−3^
Δρ_min_ = −0.27 e Å^−3^

### 

Data collection: *SMART* (Bruker, 2007 ▶); cell refinement: *SAINT* (Bruker, 2007 ▶); data reduction: *SAINT*; program(s) used to solve structure: *SHELXS97* (Sheldrick, 2008 ▶); program(s) used to refine structure: *SHELXL97* (Sheldrick, 2008 ▶); molecular graphics: *SHELXTL* (Sheldrick, 2008 ▶); software used to prepare material for publication: *SHELXTL*.

## Supplementary Material

Crystal structure: contains datablocks I, global. DOI: 10.1107/S1600536811002741/zl2345sup1.cif

Structure factors: contains datablocks I. DOI: 10.1107/S1600536811002741/zl2345Isup2.hkl

Additional supplementary materials:  crystallographic information; 3D view; checkCIF report

## Figures and Tables

**Table 1:** Hydrogen-bond geometry (Å, °)

*D*—H⋯*A*	*D*—H	H⋯*A*	*D*⋯*A*	*D*—H⋯*A*
O3*W*—H6*W*⋯O2*W*^i^	0.85	2.09	2.872 (3)	154
O3*W*—H5*W*⋯O2^ii^	0.85	2.07	2.904 (3)	165
O2*W*—H4*W*⋯O3^ii^	0.85	2.04	2.888 (3)	174
O2*W*—H3*W*⋯O1^iii^	0.85	1.96	2.812 (3)	174
O1*W*—H2*W*⋯O3*W*^iv^	0.84 (1)	1.86 (1)	2.701 (3)	178 (3)
O1*W*—H1*W*⋯O1^v^	0.84 (1)	2.33 (2)	3.096 (3)	152 (3)
O3—H3⋯O2	0.82	1.64	2.453 (2)	168
N2—H2⋯O1*W*^vi^	0.86	2.01	2.857 (3)	171

Symmetry codes: (i) 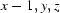; (ii) 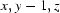; (iii) 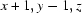; (iv) 
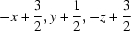
; (v) 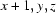; (vi) 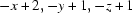.

**Fig. 1.**
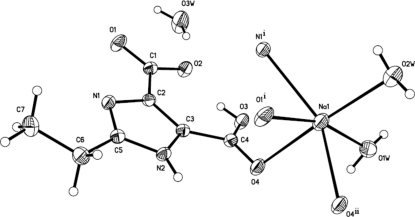
The structure of the title compound, showing the atomic numbering scheme. Non-H atoms are shown with 30% probability displacement ellipsoids. (symmetry codes: i = 1-x, 1-y, 1-z; ii = 2-x, 1-y, 1-z).

**Fig. 2.**
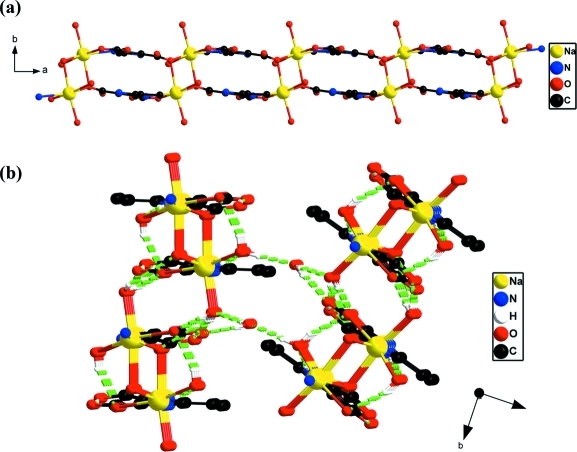
(a) One-dimensional double chain constructed of Na2O2 subunits and H2EIDC ligands propagating along the a axis (H atoms are omitted for clarity); (b) A view of the three-dimensional network constructed by O—H···O and N—H···O hydrogen bonding interactions (H atoms not involved in the hydrogen bonds are omitted for clarity).

## supplementary crystallographic information

### Comment

The rational design and synthesis of novel metal-coordination complexes *via* deliberate selection of metal ions and organic ligands has attracted much attention due to the fascinating structures that can be obtained and their potential applications in catalysis, magnetism, photoluminescence and gas storage (Sava *et al.*,2009; Lu *et al.*, 2010; Xue *et al.*, 2009). The 4,5-imidazoledicarboxylic acid (H_3_IDC) ligand exhibits flexible multi-functional coordination sites involving two N atoms of the imidazole ring and four carboxyl O atoms, and has been widely used to construct novel supramolecular architectures (Zou *et al.*, 2006; Li *et al.*, 2006; Sun *et al.*, 2005). To augment the data for the well studied H_3_IDC ligand, we recently chose to study a closely related ligand, 2-ethyl-1*H*-imidazole-4,5-dicarboxylic acid (H_3_EIDC) with an ethyl substitutent in the 2-position of the imidazole group, which could be a good candidate for generating intriguing supramolecular networks. To the best of our knowledge, only a few coordination polymers based on the H_3_EIDC ligand have been reported so far (Wang *et al.*, 2008; Zhang *et al.*, 2010). We report herein the hydrothermal synthesis and crystal structure of a new Na^I^ complex, the title compound.

As illustrated in Fig. 1, the title complex, [Na(C_7_H_7_N_2_O_4_)_2_(H_2_O)_2_].H_2_O, comprises one H_2_EIDC ligand, one Na^I^ ion, two coordinated water molecules and one solvent water molecule. Each Na^I^ cation exhibits a distorted octahedral geometry and is six-coordinated by three oxygen (O4, O1^i^ and O4^ii^) atoms and one nitrogen (N^i^) atom of three distinct H_2_EIDC ligands and two oxygen atoms (O1W and O2W) from two coordinated water molecules (symmetry codes: i = 1-*x*, 1-*y*, 1-*z*; ii = 2-*x*, 1-*y*, 1-*z*). The equatorial plane is built by the O4, O1^i^, O1W and N1^i^ atoms and the apical positions are occupied by O2W and O4^ii^. Two adjacent Na centers are bridged by two carboxyl oxygen atoms to form a Na_2_O_2_ subunit with a Na—Na distance of 3.684 (2) Å, and the Na_2_O_2_ subunits are linked by H_2_EIDC ligands to generate a one-dimensional double chain propagating along the *a* axis (Fig. 2a). The adjacent one-dimensional chains are connected into a three-dimensional supramolecular structure (Fig. 2 b) *via* N—H···O and O—H···O hydrogen bonds involving the uncoordinated imidazole N atoms, the uncoordinated and coordinated carboxylate O atoms from the H_2_EIDC ligands and the uncoordinated and coordinated water molecules (Table 1).

### Experimental

A mixture of NaOH (0.1 mmol, 0.004 g) and 2-ethyl-1*H*-imidazole-4,5-dicarboxylic acid (0.5 mmol, 0.9 g) in 10 ml of H_2_O was sealed in an autoclave equipped with a Teflon liner (20 ml) and then heated to 433 K for 4 days. Colorless crystals were obtained by slow evaporation of the solvent at room temperature with a yield of 42% based on NaOH.

### Refinement

H atoms of the water molecule were located in a difference Fourier map and refined as riding with an O—H distance restraint of 0.84 (1) Å, with *U*_iso_(H) = 1.5 *U*_eq_. The H···H distances within the water molecules were restraint to 1.39 (1) Å. Carboxyl H atoms were located in a difference map but were refined as riding on the parent O atoms with O—H = 0.82 Å and *U*_iso_(H) = 1.5 *U*_eq_(O). Carbon and nitrogen bound H atoms were placed at calculated positions and were treated as riding on the parent C or N atoms with C—H = 0.96 (methyl), 0.97 (methylene) and N—H = 0.86 Å, *U*_iso_(H) = 1.2 or 1.5 *U*_eq_(C, N).

### Crystal data

**Table:**

[Na(C_7_H_7_N_2_O_4_)(H_2_O)_2_]·H_2_O	*F*(000) = 544
*M**_r_* = 260.18	*D*_x_ = 1.527 Mg m^−^^3^
Monoclinic, *P*2_1_/*n*	Mo *K*α radiation, λ = 0.71073 Å
Hall symbol: -P 2yn	Cell parameters from 1702 reflections
*a* = 8.5231 (8) Å	θ = 2.5–25.9°
*b* = 7.0598 (7) Å	µ = 0.17 mm^−^^1^
*c* = 19.0329 (17) Å	*T* = 298 K
β = 98.880 (1)°	Block, colorless
*V* = 1131.51 (18) Å^3^	0.49 × 0.48 × 0.34 mm
*Z* = 4	

### Data collection

**Table:**

Bruker SMART 1000 CCD area-detector diffractometer	1991 independent reflections
Radiation source: fine-focus sealed tube	1549 reflections with *I* > 2σ(*I*)
graphite	*R*_int_ = 0.043
φ and ω scans	θ_max_ = 25.0°, θ_min_ = 2.5°
Absorption correction: multi-scan (*SADABS*; Bruker, 2007)	*h* = −6→10
*T*_min_ = 0.923, *T*_max_ = 0.946	*k* = −8→8
5410 measured reflections	*l* = −22→21

### Refinement

**Table:**

Refinement on *F*^2^	Secondary atom site location: difference Fourier map
Least-squares matrix: full	Hydrogen site location: inferred from neighbouring sites
*R*[*F*^2^ > 2σ(*F*^2^)] = 0.039	H atoms treated by a mixture of independent and constrained refinement
*wR*(*F*^2^) = 0.109	*w* = 1/[σ^2^(*F*_o_^2^) + (0.0431*P*)^2^ + 0.658*P*] where *P* = (*F*_o_^2^ + 2*F*_c_^2^)/3
*S* = 1.04	(Δ/σ)_max_ < 0.001
1991 reflections	Δρ_max_ = 0.33 e Å^−^^3^
162 parameters	Δρ_min_ = −0.27 e Å^−^^3^
9 restraints	Extinction correction: *SHELXL97* (Sheldrick, 2008), Fc^*^=kFc[1+0.001xFc^2^λ^3^/sin(2θ)]^-1/4^
Primary atom site location: structure-invariant direct methods	Extinction coefficient: 0.116 (7)

### Special details

**Table:**

Geometry. All e.s.d.'s (except the e.s.d. in the dihedral angle between two l.s. planes) are estimated using the full covariance matrix. The cell e.s.d.'s are taken into account individually in the estimation of e.s.d.'s in distances, angles and torsion angles; correlations between e.s.d.'s in cell parameters are only used when they are defined by crystal symmetry. An approximate (isotropic) treatment of cell e.s.d.'s is used for estimating e.s.d.'s involving l.s. planes.
Refinement. Refinement of *F*^2^ against ALL reflections. The weighted *R*-factor *wR* and goodness of fit *S* are based on *F*^2^, conventional *R*-factors *R* are based on *F*, with *F* set to zero for negative *F*^2^. The threshold expression of *F*^2^ > σ(*F*^2^) is used only for calculating *R*-factors(gt) *etc*. and is not relevant to the choice of reflections for refinement. *R*-factors based on *F*^2^ are statistically about twice as large as those based on *F*, and *R*- factors based on ALL data will be even larger.

### Fractional atomic coordinates and isotropic or equivalent isotropic displacement parameters (Å^2^)

**Table:**

	*x*	*y*	*z*	*U*_iso_*/*U*_eq_	
Na1	0.93820 (11)	0.32398 (16)	0.56175 (5)	0.0388 (4)	
N1	0.3469 (2)	0.7014 (3)	0.42293 (10)	0.0288 (5)	
N2	0.5987 (2)	0.6488 (3)	0.41437 (10)	0.0283 (5)	
H2	0.6816	0.6165	0.3966	0.034*	
O1	0.2192 (2)	0.8220 (3)	0.54163 (10)	0.0413 (5)	
O2	0.4583 (2)	0.8601 (3)	0.60539 (9)	0.0361 (5)	
O3	0.7371 (2)	0.8029 (3)	0.59418 (9)	0.0361 (5)	
H3	0.6453	0.8360	0.5953	0.054*	
O4	0.8713 (2)	0.6644 (3)	0.51722 (9)	0.0374 (5)	
O1W	1.1322 (2)	0.5012 (3)	0.63961 (10)	0.0413 (5)	
H1W	1.132 (4)	0.608 (2)	0.6204 (13)	0.062*	
H2W	1.152 (4)	0.509 (4)	0.6843 (6)	0.062*	
O2W	1.0214 (2)	0.0314 (3)	0.61812 (10)	0.0444 (6)	
H3W	1.0861	−0.0263	0.5958	0.067*	
H4W	0.9347	−0.0303	0.6128	0.067*	
O3W	0.3117 (3)	0.0343 (4)	0.71733 (11)	0.0803 (9)	
H5W	0.3704	−0.0161	0.6902	0.120*	
H6W	0.2135	0.0274	0.7005	0.120*	
C1	0.3656 (3)	0.8138 (4)	0.54743 (13)	0.0292 (6)	
C2	0.4401 (3)	0.7466 (3)	0.48677 (12)	0.0256 (6)	
C3	0.5973 (3)	0.7131 (3)	0.48215 (12)	0.0255 (6)	
C4	0.7464 (3)	0.7262 (4)	0.53360 (13)	0.0277 (6)	
C5	0.4475 (3)	0.6448 (4)	0.38032 (13)	0.0280 (6)	
C6	0.4053 (3)	0.5841 (5)	0.30430 (13)	0.0391 (7)	
H6A	0.4522	0.4610	0.2986	0.047*	
H6B	0.4512	0.6731	0.2744	0.047*	
C7	0.2284 (3)	0.5722 (5)	0.27913 (15)	0.0472 (8)	
H7A	0.1832	0.4770	0.3059	0.071*	
H7B	0.2094	0.5397	0.2296	0.071*	
H7C	0.1804	0.6925	0.2859	0.071*	

### Atomic displacement parameters (Å^2^)

**Table:**

	*U*^11^	*U*^22^	*U*^33^	*U*^12^	*U*^13^	*U*^23^
Na1	0.0261 (6)	0.0525 (8)	0.0381 (6)	0.0021 (5)	0.0057 (4)	−0.0010 (5)
N1	0.0239 (11)	0.0336 (12)	0.0288 (11)	0.0004 (9)	0.0038 (9)	0.0000 (9)
N2	0.0228 (11)	0.0363 (13)	0.0270 (11)	0.0012 (9)	0.0083 (8)	−0.0012 (9)
O1	0.0241 (10)	0.0583 (13)	0.0433 (11)	0.0016 (9)	0.0107 (8)	−0.0118 (10)
O2	0.0295 (10)	0.0503 (12)	0.0289 (10)	0.0009 (8)	0.0057 (7)	−0.0098 (8)
O3	0.0243 (9)	0.0520 (13)	0.0319 (10)	0.0009 (8)	0.0033 (7)	−0.0077 (9)
O4	0.0229 (10)	0.0517 (13)	0.0379 (10)	0.0049 (8)	0.0061 (8)	−0.0022 (9)
O1W	0.0422 (11)	0.0494 (13)	0.0332 (10)	0.0032 (10)	0.0086 (9)	−0.0007 (9)
O2W	0.0342 (10)	0.0526 (13)	0.0464 (12)	0.0014 (9)	0.0062 (8)	−0.0099 (10)
O3W	0.0568 (15)	0.148 (3)	0.0359 (12)	0.0132 (16)	0.0053 (10)	−0.0115 (15)
C1	0.0279 (14)	0.0297 (14)	0.0312 (14)	−0.0002 (11)	0.0082 (11)	0.0000 (11)
C2	0.0246 (12)	0.0257 (13)	0.0272 (12)	−0.0007 (10)	0.0056 (10)	0.0015 (10)
C3	0.0257 (13)	0.0260 (13)	0.0255 (12)	−0.0001 (10)	0.0060 (10)	0.0000 (10)
C4	0.0255 (13)	0.0291 (14)	0.0293 (13)	−0.0002 (11)	0.0062 (10)	0.0011 (11)
C5	0.0266 (13)	0.0307 (14)	0.0272 (13)	0.0003 (10)	0.0054 (10)	0.0006 (11)
C6	0.0391 (16)	0.0506 (19)	0.0275 (14)	−0.0012 (13)	0.0048 (11)	−0.0024 (13)
C7	0.0446 (17)	0.058 (2)	0.0351 (15)	−0.0049 (15)	−0.0052 (12)	0.0008 (14)

### Geometric parameters (Å, °)

**Table:**

Na1—O4^i^	2.378 (2)	O4—Na1^i^	2.378 (2)
Na1—O2W	2.384 (2)	O1W—H1W	0.840 (11)
Na1—O1W	2.396 (2)	O1W—H2W	0.843 (11)
Na1—O1^ii^	2.433 (2)	O2W—H3W	0.8500
Na1—N1^ii^	2.498 (2)	O2W—H4W	0.8500
Na1—O4	2.583 (2)	O3W—H5W	0.8500
N1—C5	1.329 (3)	O3W—H6W	0.8499
N1—C2	1.383 (3)	C1—C2	1.480 (3)
N1—Na1^ii^	2.498 (2)	C2—C3	1.377 (3)
N2—C5	1.351 (3)	C3—C4	1.482 (3)
N2—C3	1.369 (3)	C5—C6	1.499 (3)
N2—H2	0.8600	C6—C7	1.512 (4)
O1—C1	1.237 (3)	C6—H6A	0.9700
O1—Na1^ii^	2.432 (2)	C6—H6B	0.9700
O2—C1	1.296 (3)	C7—H7A	0.9600
O3—C4	1.287 (3)	C7—H7B	0.9600
O3—H3	0.8200	C7—H7C	0.9600
O4—C4	1.234 (3)		
			
O4^i^—Na1—O2W	97.45 (7)	Na1—O1W—H2W	132 (2)
O4^i^—Na1—O1W	84.24 (7)	H1W—O1W—H2W	111.4 (15)
O2W—Na1—O1W	92.58 (7)	Na1—O2W—H3W	111.2
O4^i^—Na1—O1^ii^	81.27 (7)	Na1—O2W—H4W	101.4
O2W—Na1—O1^ii^	94.91 (8)	H3W—O2W—H4W	108.2
O1W—Na1—O1^ii^	164.44 (8)	H5W—O3W—H6W	112.6
O4^i^—Na1—N1^ii^	147.95 (8)	O1—C1—O2	122.6 (2)
O2W—Na1—N1^ii^	96.45 (8)	O1—C1—C2	119.6 (2)
O1W—Na1—N1^ii^	123.79 (8)	O2—C1—C2	117.8 (2)
O1^ii^—Na1—N1^ii^	68.86 (7)	C3—C2—N1	109.7 (2)
O4^i^—Na1—O4	84.16 (7)	C3—C2—C1	130.1 (2)
O2W—Na1—O4	171.50 (8)	N1—C2—C1	120.1 (2)
O1W—Na1—O4	79.24 (7)	N2—C3—C2	105.48 (19)
O1^ii^—Na1—O4	93.59 (7)	N2—C3—C4	120.8 (2)
N1^ii^—Na1—O4	86.31 (7)	C2—C3—C4	133.7 (2)
O4^i^—Na1—Na1^i^	44.22 (5)	O4—C4—O3	123.3 (2)
O2W—Na1—Na1^i^	140.98 (7)	O4—C4—C3	119.7 (2)
O1W—Na1—Na1^i^	78.72 (6)	O3—C4—C3	116.9 (2)
O1^ii^—Na1—Na1^i^	86.90 (6)	N1—C5—N2	111.0 (2)
N1^ii^—Na1—Na1^i^	120.13 (7)	N1—C5—C6	126.4 (2)
O4—Na1—Na1^i^	39.94 (4)	N2—C5—C6	122.6 (2)
C5—N1—C2	105.55 (19)	C5—C6—C7	113.6 (2)
C5—N1—Na1^ii^	141.28 (17)	C5—C6—H6A	108.8
C2—N1—Na1^ii^	110.58 (15)	C7—C6—H6A	108.8
C5—N2—C3	108.2 (2)	C5—C6—H6B	108.8
C5—N2—H2	125.9	C7—C6—H6B	108.8
C3—N2—H2	125.9	H6A—C6—H6B	107.7
C1—O1—Na1^ii^	118.37 (16)	C6—C7—H7A	109.5
C4—O3—H3	109.5	C6—C7—H7B	109.5
C4—O4—Na1^i^	147.92 (17)	H7A—C7—H7B	109.5
C4—O4—Na1	113.65 (16)	C6—C7—H7C	109.5
Na1^i^—O4—Na1	95.84 (7)	H7A—C7—H7C	109.5
Na1—O1W—H1W	104 (2)	H7B—C7—H7C	109.5
			
O4^i^—Na1—O4—C4	167.0 (2)	N1—C2—C3—N2	0.6 (3)
O1W—Na1—O4—C4	−107.73 (17)	C1—C2—C3—N2	178.7 (2)
O1^ii^—Na1—O4—C4	86.20 (17)	N1—C2—C3—C4	−176.6 (3)
N1^ii^—Na1—O4—C4	17.68 (17)	C1—C2—C3—C4	1.5 (5)
Na1^i^—Na1—O4—C4	167.0 (2)	Na1^i^—O4—C4—O3	−121.0 (3)
O4^i^—Na1—O4—Na1^i^	0.0	Na1—O4—C4—O3	83.8 (3)
O1W—Na1—O4—Na1^i^	85.23 (7)	Na1^i^—O4—C4—C3	59.2 (4)
O1^ii^—Na1—O4—Na1^i^	−80.84 (7)	Na1—O4—C4—C3	−96.0 (2)
N1^ii^—Na1—O4—Na1^i^	−149.36 (8)	N2—C3—C4—O4	−5.9 (4)
Na1^ii^—O1—C1—O2	−168.77 (19)	C2—C3—C4—O4	171.0 (3)
Na1^ii^—O1—C1—C2	10.4 (3)	N2—C3—C4—O3	174.3 (2)
C5—N1—C2—C3	−1.1 (3)	C2—C3—C4—O3	−8.9 (4)
Na1^ii^—N1—C2—C3	164.78 (16)	C2—N1—C5—N2	1.1 (3)
C5—N1—C2—C1	−179.3 (2)	Na1^ii^—N1—C5—N2	−157.43 (19)
Na1^ii^—N1—C2—C1	−13.5 (3)	C2—N1—C5—C6	−178.6 (3)
O1—C1—C2—C3	−175.0 (3)	Na1^ii^—N1—C5—C6	22.8 (5)
O2—C1—C2—C3	4.3 (4)	C3—N2—C5—N1	−0.7 (3)
O1—C1—C2—N1	2.9 (4)	C3—N2—C5—C6	179.0 (2)
O2—C1—C2—N1	−177.8 (2)	N1—C5—C6—C7	−5.9 (4)
C5—N2—C3—C2	0.0 (3)	N2—C5—C6—C7	174.4 (2)
C5—N2—C3—C4	177.7 (2)		

Symmetry codes: (i) −*x*+2, −*y*+1, −*z*+1; (ii) −*x*+1, −*y*+1, −*z*+1.

### Hydrogen-bond geometry (Å, °)

**Table:**

*D*—H···*A*	*D*—H	H···*A*	*D*···*A*	*D*—H···*A*
O3W—H6W···O2W^iii^	0.85	2.09	2.872 (3)	154
O3W—H5W···O2^iv^	0.85	2.07	2.904 (3)	165
O2W—H4W···O3^iv^	0.85	2.04	2.888 (3)	174
O2W—H3W···O1^v^	0.85	1.96	2.812 (3)	174
O1W—H2W···O3W^vi^	0.84 (1)	1.86 (1)	2.701 (3)	178 (3)
O1W—H1W···O1^vii^	0.84 (1)	2.33 (2)	3.096 (3)	152 (3)
O3—H3···O2	0.82	1.64	2.453 (2)	168
N2—H2···O1W^i^	0.86	2.01	2.857 (3)	171

Symmetry codes: (iii) *x*−1, *y*, *z*; (iv) *x*, *y*−1, *z*; (v) *x*+1, *y*−1, *z*; (vi) −*x*+3/2, *y*+1/2, −*z*+3/2; (vii) *x*+1, *y*, *z*; (i) −*x*+2, −*y*+1, −*z*+1.

## References

[bb1] Bruker (2007). *SMART*, *SAINT* and *SADABS* Bruker AXS Inc., Madison, Wisconsin, USA.

[bb2] Li, C. J., Hu, S., Li, W., Lam, C. K., Zheng, Y. Z. & Tong, M. L. (2006). *Eur. J. Inorg. Chem.* pp. 1931–1935.

[bb3] Lu, J., Ting, H., Zhang, X. X., Wang, D. Q. & Niu, M. J. (2010). *Z. Anorg. Allg. Chem.* **636**, 641–647.

[bb4] Sava, D. F., Kravtsov, V. C., Eckert, J., Eubank, J. F., Nouar, F. & Eddaoudi, M. (2009). *J. Am. Chem. Soc.* **131**, 10394–10396. 10.1021/ja903287v 19588971

[bb5] Sheldrick, G. M. (2008). *Acta Cryst.* A**64**, 112–122. 10.1107/S0108767307043930 18156677

[bb6] Sun, Y. Q., Zhang, J., Chen, Y. M. & Yang, G. Y. (2005). *Angew. Chem. Int. Ed.* **44**, 5814–5817. 10.1002/anie.200500453 16059952

[bb7] Wang, S., Zhang, L. R., Li, G. H., Huo, Q. S. & Liu, Y. L. (2008). *CrystEngComm*, **10**, 1662–1666.

[bb8] Xue, M., Zhu, G. S., Ding, H., Wu, L., Zhao, X. J., Jin, Z. & Qiu, S. L. (2009). *Cryst. Growth Des.* **9**, 1481–1488.

[bb9] Zhang, F. W., Li, Z. F., Ge, T. Z., Yao, H. C., Li, G., Lu, H. J. & Zhu, Y. Y. (2010). *Inorg. Chem.* **49**, 3776–3788. 10.1021/ic902483m 20230024

[bb10] Zou, R. Q., Sakurai, H. & Xu, Q. (2006). *Angew. Chem. Int. Ed.* **45**, 2542–2546. 10.1002/anie.200503923 16544346

